# Viraemic-time predicts mortality among people living with HIV on second-line antiretroviral treatment in Myanmar: A retrospective cohort study

**DOI:** 10.1371/journal.pone.0271910

**Published:** 2022-07-29

**Authors:** Anita Mesic, Tom Decroo, Htay Thet Mar, Bart K. M. Jacobs, Moe Pyae Thandar, Thin Thin Thwe, Aung Aung Kyaw, Mitchell Sangma, David Beversluis, Elkin Bermudez-Aza, Alexander Spina, Darli Po Po Aung, Erwan Piriou, Koert Ritmeijer, Josefien Van Olmen, Htun Nyunt Oo, Lutgarde Lynen

**Affiliations:** 1 Public Health Department, Médecins Sans Frontières, Amsterdam, The Netherlands; 2 Department of Clinical Sciences, Institute of Tropical Medicine, Antwerp, Belgium; 3 Research Foundation Flanders, Brussels, Belgium; 4 Medical Department, Médecins Sans Frontières, Yangon, Myanmar; 5 University of Exeter Medical School, Exeter, United Kingdom; 6 Fondation Mérieux, Yangon, Myanmar; 7 Department of Family Medicine and Population Health, University of Antwerp, Antwerpen, Belgium; 8 National AIDS Programme, Ministry of Health and Sport, Naypyidaw, Myanmar; University of Sassari, ITALY

## Abstract

**Introduction:**

Despite HIV viral load (VL) monitoring being serial, most studies use a cross-sectional design to evaluate the virological status of a cohort. The objective of our study was to use a simplified approach to calculate viraemic-time: the proportion of follow-up time with unsuppressed VL above the limit of detection. We estimated risk factors for higher viraemic-time and whether viraemic-time predicted mortality in a second-line antiretroviral treatment (ART) cohort in Myanmar.

**Methods:**

We conducted a retrospective cohort analysis of people living with HIV (PLHIV) who received second-line ART for a period >6 months and who had at least two HIV VL test results between 01 January 2014 and 30 April 2018. Fractional logistic regression assessed risk factors for having higher viraemic-time and Cox proportional hazards regression assessed the association between viraemic-time and mortality. Kaplan-Meier curves were plotted to illustrate survival probability for different viraemic-time categories.

**Results:**

Among 1,352 participants, 815 (60.3%) never experienced viraemia, and 172 (12.7%), 214 (15.8%), and 80 (5.9%) participants were viraemic <20%, 20–49%, and 50–79% of their total follow-up time, respectively. Few (71; 5.3%) participants were ≥80% of their total follow-up time viraemic. The odds for having higher viraemic-time were higher among people with a history of injecting drug use (aOR 2.01, 95% CI 1.30–3.10, p = 0.002), sex workers (aOR 2.10, 95% CI 1.11–4.00, p = 0.02) and patients treated with lopinavir/ritonavir (vs. atazanavir; aOR 1.53, 95% CI 1.12–2.10, p = 0.008). Viraemic-time was strongly associated with mortality hazard among those with 50–79% and ≥80% viraemic-time (aHR 2.92, 95% CI 1.21–7.10, p = 0.02 and aHR 2.71, 95% CI 1.22–6.01, p = 0.01). This association was not observed in those with viraemic-time <50%.

**Conclusions:**

Key populations were at risk for having a higher viraemic-time on second-line ART. Viraemic-time predicts clinical outcomes. Differentiated services should target subgroups at risk for a higher viraemic-time to control both HIV transmission and mortality.

## Introduction

An increasing number of treatment-experienced people living with HIV (PLHIV) require second-line or third-line antiretroviral treatment (ART) regimens. It has been estimated that by 2030, about 4.6 million people in Sub-Saharan Africa alone will receive second-line ART [1]. For the majority of adult PLHIV, second-line ART includes two nucleoside (nucleotide) reverse transcriptase inhibitors (NRTIs) combined with a ritonavir-boosted protease inhibitor (PI): atazanavir or lopinavir.

World Health Organization (WHO) guidelines recommend routine monitoring of ART effectiveness and adherence to treatment by performing HIV viral load (VL) testing at 6 months after treatment initiation and then every subsequent 12 months [2]. In resource-limited settings, access to routine HIV VL testing is still suboptimal due to the cost and often also due to a lack of awareness among healthcare providers and patients about the benefits of regular VL monitoring [3]. Coverage of VL testing and access to second- and third-line ART for PLHIV with virological failure remain challenging in many high burden settings [4–6]. Globally, at the end of 2019, UNAIDS estimated that 59% (95% CI 49–69%) of all PLHIV achieved virological suppression. The UNAIDS target of 95% virological suppression among all those on ART by 2030 seems yet out of reach [7]. Virological suppression through highly effective ART is an important component of prevention of sexual transmission of HIV [8–12]. Since 2016, The Prevention Access Campaign with the slogan “Undetectable = Untransmittable” (“U = U”) has been essential in shaping public opinion, fighting stigma, and reducing future HIV infections [13]. Myanmar had an estimated 220,000 PLHIV and 77% ART coverage at the end of 2019 [14]. Despite substantially improved access to HIV care, in 2019 only 72% of PLHIV on ART had access to VL monitoring [15]. Médecins Sans Frontières (MSF) has been providing HIV care in Myanmar in close collaboration with the National AIDS Programme (NAP) since 2003. The second-line ART cohort studied here is the largest and the oldest cohort with long-term access to routine virological monitoring in Myanmar.

Despite serial HIV VL monitoring being implemented in many HIV cohorts, most of the studies use a cross-sectional design to evaluate virological status. However, during HIV treatment, especially when taking second-line treatment with a high genetic barrier [16, 17], patients may transition back and forth between different virological states (suppressed vs. unsuppressed). By overlooking these transitions, one might over- or underestimate the overall virological suppression rates among patients on ART. In addition, cross-sectional measures do not show whether patients were suppressed or unsuppressed for a short or long fraction of their follow-up time. In 2010, Cole et al. used “viraemia copy-years” (VCY) [18] and various studies confirmed the relationship between VCY and unfavourable outcomes among PLHIV on ART [18–20]. In our second-line ART cohort, PLHIV were monitored by routine VL tests twice per year. The objective of our study was to use a simplified approach to calculate viraemic-time, defined as the proportion of cumulative unsuppressed time over follow-up time on second-line ART, and to investigate whether having higher viraemic-time predicted mortality in a second-line ART cohort in Myanmar.

## Methods

### Design and study population

We conducted a retrospective cohort analysis of PLHIV aged above 5 years who had received second-line ART for over 6 months and had at least two HIV VL test results during the study period between 01 January 2014 and 30 April 2018.

### Study setting

The study used data from the MSF HIV programme in Myanmar, which provided, free of charge, a comprehensive HIV care package to more than 58,000 PLHIV. The secondary use of programme data frequently serves operational research, which has been seen as a tool to improve outcomes of existing medical programmes or evaluate new strategies or interventions in specific contexts. Findings often inform policy change [21].

Since 2014, routine HIV VL monitoring twice a year was introduced for PLHIV on second-line ART. Different viral load assays were used during the study period: Cavidi ExaVir version 3.0 (lower limit of detection, LLD, 200 copies/mL) and Xpert® HIV-1 Viral Load

(LLD 40 copies/mL). Management of virological failure in Myanmar relies on a public health approach [2]. For PLHIV with detectable plasma viraemia ≥1000 copies/mL, the national guideline recommends enhanced adherence counselling (EAC), which includes identifying barriers to adherence and providing intensified psychosocial support to sustain long-term compliance [22]. In case of virological failure while on second-line ART, the switch to third-line ART is often delayed. Reasons for failure include suboptimal adherence and HIV drug resistance; only the latter is a definite indication for switching to third-line ART.

### Study variables

**Fig 1:**
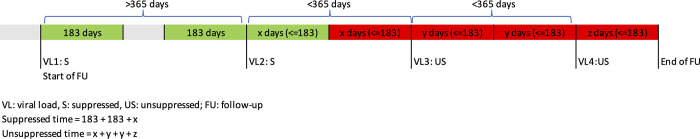
Example of allocation of unsuppressed and suppressed time. Cumulative unsuppressed or suppressed time was the time during which we assumed that a patient had HIV VL above or below the limit of detection, respectively. Follow-up time was defined as a sum of cumulative unsuppressed and suppressed time and started on the date of the first VL test and ended on the date of the last VL test plus 183 days. When two consecutive VL tests showed the same result, either viraemia or suppression, the time in between both tests was considered unsuppressed or suppressed. When two consecutive VL tests showed different results, the time in between both tests was divided in half, adding both unsuppressed and suppressed time. When the time between two VL tests was more than 365 days, a maximum of 183 days was added to either unsuppressed or suppressed time.

The study used routine programme data collected from standardised patient forms and encoded in the MSF HIV programme database, FUCHIA (Follow-up and Care of HIV Infection and AIDS). To calculate the viraemic-time, we divided the cumulative unsuppressed time by follow-up time (both in days). Cumulative unsuppressed time was the time during which we assumed that a patient had plasma HIV VL > 200 copies/mL (unsuppressed viral load). Cumulative suppressed time was the time during which we assumed that a patient had undetectable (suppressed) HIV VL. Follow-up time was defined as a sum of cumulative unsuppressed and suppressed time. When two consecutive VL tests showed the same result, either viraemia or suppression, the time in between both tests was considered unsuppressed or suppressed, respectively. When two consecutive VL tests showed different results, the time in between both tests was divided in half, adding both unsuppressed and suppressed time. Patients were supposed to have VL tests twice a year; however, if the time between two VL tests was more than 365 days, a maximum of 183 days was added to either unsuppressed or suppressed time (Fig 1). Follow-up time started on the date of the first VL test and ended on the date of the last VL test plus a maximum of 183 days, to ensure that each VL result was included.

**Fig 2:**
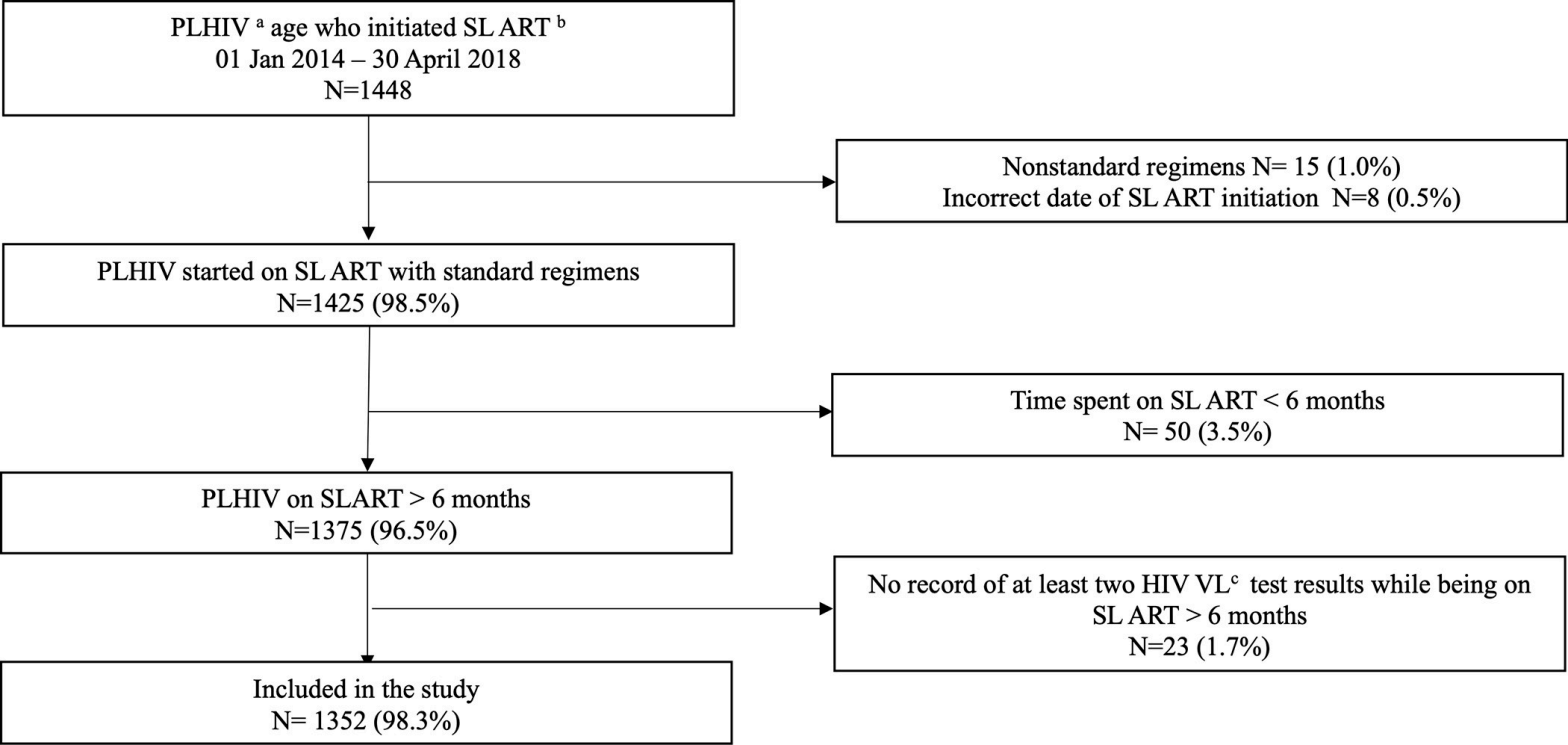
Flowchart for inclusion of participants in the study. ^a^PLHIV: People living with HIV, ^b^SL ART: Second-line antiretroviral treatment, ^c^ HIV VL: HIV viral load.

For assessing the mortality hazard during second-line ART, observation time was calculated as the time between the date of the second VL test and the date of the primary outcome (death). Follow-up started on the date of the second VL test to avoid immortal time bias [23] since those without two VL tests were excluded from the analysis (Fig 2). For patients that did not experience the outcome, censoring occurred on the dates of transfer out (to the NAP), switch to third-line ART, or the last visit during the study period.

### Data analysis

The normal distribution of continuous variables was assessed by Skewness/Kurtosis tests for Normality and plotting histograms for each of the continuous variables. When the distribution was not normal, we calculated medians and interquartile ranges (IQR).

To calculate the odds ratios (OR) and respective 95% confidence intervals (CI) for having an unsuppressed VL we performed a fractional logistic regression. To compute hazard ratios (HR) and respective 95% CI for mortality we performed a Cox proportional hazards regression. For both regressions, forward stepwise selection was used to construct the final multivariable model. First, variables known for their clinical importance (age, gender) were included, regardless of their association with the outcome. Additional variables were stepwise included in the multivariable model if a) they were associated with outcome (p<0.10) in the univariate analysis, and b) if they significantly improved the model (p<0.05, with Wald test for logistic regressions and likelihood ratio tests for Cox regressions). The proportional hazards assumption was tested using scaled Schoenfeld residuals. All analyses were carried out using STATA statistical software version 14.0 (STATACorp, Texas, USA).

### Ethics approval

The study was approved by the Institutional Review Board, Department of Medical Research, Ministry of Health and Sports, Myanmar (Ethics/DMR/2019/153). The study also fulfilled the exemption criteria set by the MSF independent Ethical Review Board (ERB) for *a posteriori* analyses of routinely collected clinical data—which requires patients in MSF programmes at the time of entering MSF care to have given informed written consent for secondary use of their data, including research—thus did not require MSF ERB review [24]. All medical records and data were fully anonymized before we included them in this analysis.

## Results

### Study population

During the study period, 1,448 PLHIV aged >5 years started second-line ART and 1,352 (93.4%) were included in the study (Fig 2). Their median age was 34.8 (IQR 28.4–40.6) years. The median baseline CD4 count and BMI were 162 (IQR 68–309) cells/mL and 20.5 (IQR 18.3–23.6) kg/m2, respectively. The median time on first-line and second-line ART was 49.0 (IQR 28.6–79.7) and 54.5 (IQR 44.6–65.1) months. Additional characteristics of the study population are presented in Table 1.

**Table 1:** Demographic and clinical characteristics of the study population at the time of second-line treatment initiation (N = 1,352).

Variable	Characteristics	N	%
Gender	Male	768	56.8
	Female	584	43.2
Age group (years)	5–18	224	16.5
	19–25	41	3.0
	25–45	918	68.0
	>45	169	12.5
Profession	Employed	832	61.5
	Student	49	3.6
	Unemployed	354	26.2
	Data Unavailable	117	8.7
Marital Status	Married	656	48.6
	Separated	99	7.3
	Single	421	31.1
	Widow	129	9.5
	Data Unavailable	47	3.5
Men having sex with men^a^	Yes	8	0.6
History of injecting drugs	Yes	63	4.7
History of sex work	Yes	26	1.9
Baseline CD4 (cells/mL)	<200	661	48.9
	≥200	362	26.8
	Data Unavailable	329	24.3
Baseline BMI^b^ (kg/m^2^)	<18.5	360	26.6
	≥18.5	932	69.0
	Data Unavailable	60	4.4
Baseline WHO stage	1 or 2	154	11.4
	3 or 4	1194	88.3
	Data Unavailable	4	0.3
Tuberculosis	Yes	203	15.0
Second-line regimen^c^	Atv/r-based	1134	83.9
	Lop/r-based	218	16.1
Second-line treatment duration (months)	<24	23	1.7
	24–60	842	62.3
	≥60	487	36.0
First-line treatment duration (months)	<24	274	20.3
	24–60	528	39.1
	≥60	525	38.8
	Data Unavailable^d^	25	1.8

^a^Among male population

^b^ BMI: body mass index

^c^ATV/r: Atazanavir/ritonavir, Lop/r: Lopinavir/ritonavir

^d^This group of participants started second-line ART without prior first-treatment either due to co-morbidities (tuberculosis), intolerance, or previous exposure to first-line treatment

### Viraemic-time

**Fig 3:**
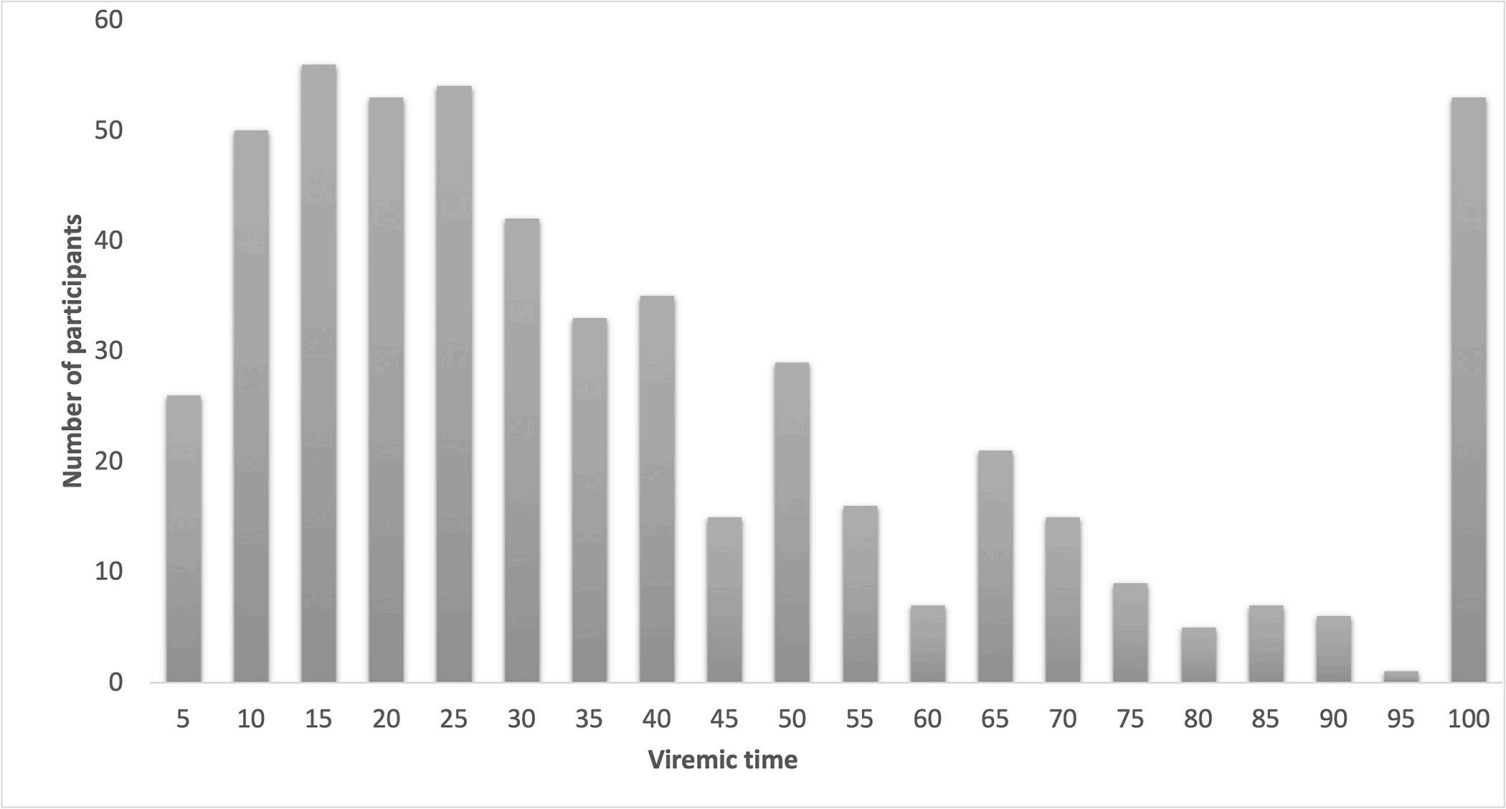
Distribution of participants by viraemic-time among those who experienced viraemia (N = 537).

There were 5,048 VL results recorded during the overall follow-up time of 1,103,817 days for 1,352 participants (median 4 VL tests per patient, IQR 2–6). In only 244 occasions, the time between two consecutive VL tests was >365 days, resulting in a reduction of 23,338 days, based on the established maximum of allocated time per VL result (365 days: with a maximum of 183 days either suppressed or unsuppressed time before and after the date of the VL test; Fig 1). As presented in Table 2, in our study population on second-line treatment, 815 (60.3%) participants never experienced viraemia, while in 172 (12.7%), 214 (15.8%), and 80 (5.9%) participants the viraemic-time was <20%, 20–49%, and 50–79% of the total follow-up time, respectively. In 71 (5.3%) participants the viraemic-time was ≥80%, of which 51 (3.8%) participants had viraemia throughout the total follow-up time (Fig 3).

**Table 2:** Proportion of time with viraemia >200 copies/mL while on second-line treatment (N = 1,352).

Viraemic-time (%)^a^	N	%	Median time on second-line ART (months)^b^	IQR^c^
No viraemia	815	60.3	54.0	44.9, 64.8
1–19	172	12.7	59.2	49.9, 69.2
20–49	214	15.8	56.8	49.3, 56.8
50–79	80	5.9	48.5	40.9, 60.4
≥80	71	5.3	42.7	32.2, 54.5

^a^ Viraemic-time is calculated as estimated time with VL >200 copies/mL over total follow-up time on second-line ART

^b^ Calculated from the start of second-line ART until the end of the study period or censoring (death, switch to third-line ART or transfer out)

^c^ IQR: Interquartile range (months)

### Risk factors for viraemia

The odds for having higher viraemic-time were higher among people with a history of injecting drug use (aOR 2.01, 95% CI 1.30–3.10, p = 0.002), sex workers (aOR 2.10, 95% CI 1.11–4.00, p = 0.02) and patients treated with a second-line lopinavir/ritonavir-based regimen (vs. atazanavir; aOR 1.53, 95% CI 1.12–2.10, p = 0.008). When compared to married participants, those who were separated had a higher odds for having higher viraemic time (aOR 1.59, 95%CI 1 01–2.38, p = 0.02). Age, gender and profession were not significantly associated with viremia in our study, as presented in Table 3.

**Table 3:** Univariable and multivariable fractional logistic regression to show the association between explanatory variables and viraemia (N = 1,352).

Variable	Category	Odds ratio (95% CI)	p-value	Adjusted Odds Ratio^a^	p-value
Gender	Female	1.17 (0.94–1.45)	0.16	1.20 (0.94–1.54)	0.15
Age		0.98 (0.97–0.99)	<0.001	0.99 (0.98–1.00)	0.41
Profession	Employed				
	Student	1.38 (0.81–2.34)	0.23	1.02 (0.57–1.81)	0.96
	Unemployed	1.22 (0.96–1.55)	0.10	0.99 (0.75–1.31)	0.95
Marital Status	Married				
	Separated	1.61(1.09–2.38)	0.02	1.59 (1.01–2.38)	0.02
	Single	1.50 (1.16–1.86)	0.002	1.31 (0.96–1.78)	0.09
	Widow	0.95 (0.63–1.43)	0.81	0.90 (0.59–1.35)	0.62
Man having sex with men	Yes	0.51 (0.18–1.46)	0.21		
History of injecting drugs	Yes	1.76 (1.16–2.66)	0.007	2.01 (1.30–3.10)	0.002
History of sex work	Yes	2.41(1.28–4.51)	0.006	2.10 (1.11–4.00)	0.02
Baseline WHO stage	1or 2				
	3 or 4	1.21 (0.86–1.71)	0.26		
Second-line regimen^b^	Atv/r^c^				
	Lop/r^c^	1.75 (1.33–2.31)	<0.001	1.53 (1.12–2.10)	0.008
Baseline tuberculosis	Yes	1.19 (0.90–1.57)	0.23		
Baseline CD4 (cells/mL)		1.00 (1.00–1.01)	0.017	1.00 (0.99–1.00)	0.10
Baseline BMI^c^ (kg/m^2^)		1.01 (0.98–1.03)	0.32		
First-line treatment duration (months)		1.00 (0.99–1.00)	0.32		

^a^ Forward stepwise selection was used to construct the final multivariable model. First, variables known for their clinical importance (age, gender) were included, regardless of their association with the outcome. Additional variables were stepwise included in the multivariable model if they were associated with outcome (p<0.10) in the univariate analysis, and b) if they significantly improved the model (p<0.05).

^b^Atv/r: atazanavir/ritonavir, Lop/r: lopinavir/ritonavir

^c^BMI: Body mass index

### Factors associated with mortality

At the end of the study period, 61 (4.5%) participants had died, 565 (41.8%) were transferred-out to the NAP, and 726 (53.7%) were still in care in the MSF programme; 5 participants had a brief history of loss to follow-up but all returned to care in the MSF programme during the study period (after a medium time of 112 days (IQR 65–145)). Twenty-two (1.63%) PLHIV switched to third-line ART during the study period. Patients with viraemic-time >50% had a higher mortality hazard (Table 4). PLHIV with viraemic-time 50–79% or >80% had an almost three-fold higher mortality hazard compared to those who were not viraemic during their follow-up time (aHR 2.92, 95% CI 1.21–7.10, p = 0.02; aHR 2.71, 95% CI 1.22–6.01, p = 0.01, respectively). Among PLHIV with viraemic-time <50%, the mortality hazard was not higher compared to those without viraemia during their follow-up period. Participants on lopinavir/ritonavir-based second-line ART had a 4.53 times higher mortality hazard compared to participants who were on an atazanavir/ritonavir-based regimen (95% CI 2.58–8.00; p<0.001). For every year increase in age, the mortality hazard increased (aHR 1.04, 95%CI 1.06–1.08, p<0.01). Gender, marital status and baseline BMI were not significantly associated with mortality in our study population (Table 4).

**Table 4:** Univariable and multivariable Cox regression to show the association between explanatory variables and mortality.

Variable	Category	Hazard ratio (95%CI)	p-value	Adjusted hazard ratio^a^ (95%CI)	p-value
Gender	Male				
	Female	1.40 (0.85–2.32)	0.19	1.44 (0.89–1.63)	0.44
Age		1.04 (1.02–1.06)	<0.001	1.06 (1.03–1.08)	<0.001
Profession	Employed				
	Student	1.15 (0.28–4.78)	0.85		
	Unemployed	1.01 (0.58–1.77)	0.97		
Marital Status	Married				
	Separated	0.67 (0.20–2.20)	0.51	0.87 (0.26–2.93)	0.82
	Single	0.80 (0.43–1.49)	0.48	1.79 (0.84–3.80)	0.13
	Widow	2.00 (1.02–3.94)	0.04	1.04 (0.49–2.24)	0.90
Man having sex with men	Yes	NA	NA		
History of injecting drugs	Yes	1.60 (0.50–5.12)	0.43		
History of sex work	Yes	1.63 (0.40–6.67)	0.50		
Baseline CD4 (cells/mL)		0.99 (0.99–1.00)	0.37		
Baseline BMI^b^ (kg/m2)		0.94 (0.88–1.00)	0.04	0.96 (0.90–1.02)	0.18
Baseline WHO stage	1 or 2				
	3 or 4	3.05 (0.75–12.50)	0.12		
Baseline Tuberculosis	Yes	1.43 (0.77–2.63)	0.25		
Second-line regimen	Atv/r^c^				
	Lop/r^c^	4.13 (2.48–6.86)	<0.001	4.53 (2.58–8.00)	<0.001
Duration of first-line treatment (months)		1.00 (0.99–1.00)	0.72		
Viraemic-time (%)	0				
	1–19	0.53 (0.21–1.38)	0.20	0.60 (0.23–1.58)	0.30
	20–49	0.76 (0.34–1.73)	0.51	0.88 (0.38–2.04)	0.78
	50–79	3.06 (1.41–6.67)	0.005	2.92 (1.21–7.10)	0.02
	≥80	5.05 (2.46–10.39)	<0.001	2.71 (1.22–6.01)	0.01

^a^ Forward stepwise selection was used to construct the final multivariable model. First, variables known for their clinical importance (age, gender) were included, regardless of their association with the outcome. Additional variables were stepwise included in the multivariable model if they were associated with outcome (p<0.10) in the univariate analysis, and b) if they significantly improved the model (p<0.05)

^b^BMI: Body mass index

^c^Atv/r: atazanavir/ritonavir, Lop/r: lopinavir/ritonavir

**Fig 4:**
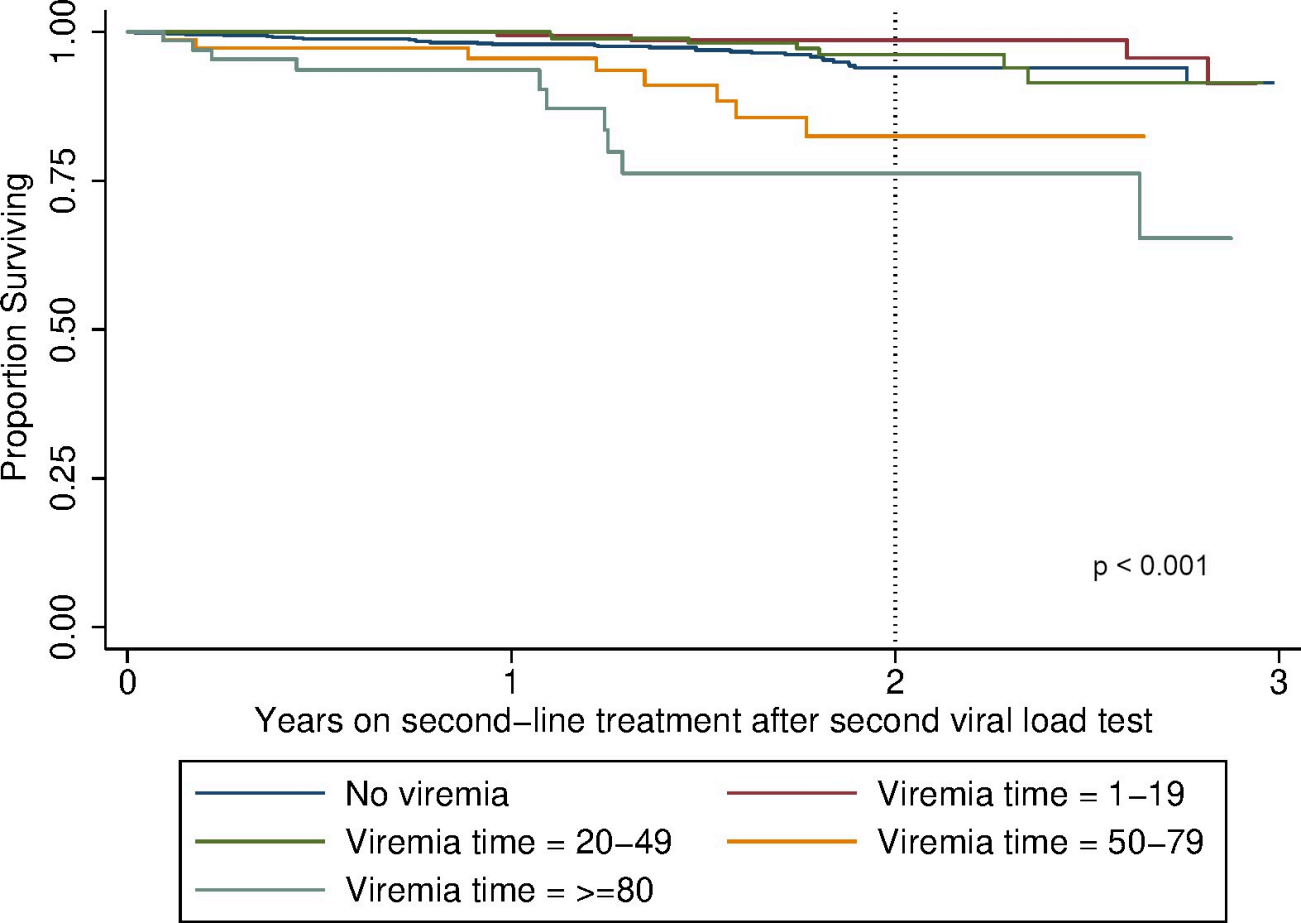
Kaplan-Meier survival estimates by viraemic-time.

The cumulative mortality hazard (Fig 4) at two years of follow-up on second-line treatment since the second VL was 6.2% (95% CI 4.2–9.1%) for PLHIV with no viraemic-time, and 1.3% (95% CI 0.3–5.5), 3.8% (95% CI 1.5–9.5%), 19.01% (95% CI 9.2–39.6%) and 26.7% (95% CI 13.2–54.9%) for those with 1–19%, 20–49%, 50–79%, or **≥**80% viraemic-time (p<0.001), respectively.

## Discussion

The study of our second-line ART cohort in Myanmar demonstrated a close to three-fold increase in mortality hazard in PLHIV who were more than half of their follow-up time viraemic, after controlling for other relevant factors. Those who were viraemic, but less than half of their follow-up time, were not at risk of mortality. HIV is a chronic disease. Being viraemic does not equal immediate symptomatic morbidity and this may explain why mortality increases only when patients are viraemic for a longer period of time [25]. Studies show that treatment interruptions might lead to a drop in CD4 counts, increased incidence of opportunistic infections, and mortality [26], while others argue that guided treatment interruptions might be a successful strategy in long-term HIV care [27]. This practice may work—without resulting in an increased mortality risk—as long as the immune system remains intact, and no resistance is acquired to the prescribed ART drugs. However, this has important implications since transmission is not halted. Multiple studies have shown that the risk of transmission increases with increased HIV VL [28–30]. We speculate that those who were viraemic, and not at risk of dying, continued with their daily life and could contribute to HIV transmission in the community, especially when their HIV VL >1000 copies/mL [31].

As a measure for unsuppressed VL, we used any viraemia that was above 200 cells/mL. The WHO now recommends intensive adherence support and three-monthly VL monitoring for PLHIV with detectable viraemia <1000 copies/mL [32] and there is an increasing amount of evidence on low-level viraemia being associated with unfavourable treatment outcomes [33, 34]. A recently published study from the same setting confirmed low-level viraemia as a predictor of virological failure in patients on first-line ART [35]. Further studies should evaluate which cut-off is epidemiologically and clinically the most relevant.

To calculate viraemic-time, we used a simplified approach that could be applied in programmatic settings. Multiple studies used VCY as a measure of the proportion of follow-up time with an unsuppressed VL and reported an association between VCY and having an unfavourable outcome, either morbidity [18, 36, 37] or mortality [18, 19, 38]. Further studies are needed to assess the best use of viraemic-time as a prognostic factor in routine clinical practice. Multiple studies emphasize that prolonged exposure to HIV replication leads to immune system activation [19], also involved in the pathogenesis of non-communicable diseases (NCDs) [36, 39], and increases risks of opportunistic infections [26, 40]. Viraemic-time as a predictor of morbidity was not studied in our cohort and should be subjected to further research. To successfully meet HIV control targets, 95% of all PLHIV should have a suppressed VL. Programmes measure this indicator at a given moment in time, and do not distinguish between having all PLHIV suppressed for 95% of the time or aiming at having 95% PLHIV suppressed and accepting that 5% are virologically failing until they die and “disappear” from the equation. It seems obvious that programmes aim for the best for all their patients, but the distinction cannot be made the way VL data are reported in programmatic settings. While clinicians tend to interpret VL longitudinally, usually cross-sectional designs are used to evaluate the virological response to HIV treatment within a cohort [41–43]. We believe that cumulative assessments reflect the health status and HIV control of the entire cohort better than cross-sectionally measured indicators.

The risk of having higher viraemic-time was double in people with a history of injecting drug use or sex work. Key populations are disproportionately affected by the HIV epidemic and are known to play a fundamental role in HIV transmission in many settings, including Myanmar [44]. In 2019, the estimated HIV prevalence among people who inject drugs, sex workers, and men having sex with men in Myanmar was 28.5%, 25.0%, and 20.0%, respectively [14]. In addition to poor access to care, non-sustained suppression can partially explain the high HIV incidence among those populations [45]. In the context of the present study, key populations are often mobile, and their working hours or practices impede easy access to care. Additionally, stigma, discrimination, and criminalisation of their practices interfere with adherence, thus increase viraemic-time and consequently results in HIV transmission. To address the specific needs of key populations, differentiated service delivery models, including a comprehensive package of preventative and treatment services have been recommended to achieve “treatment as prevention” [46]. To simultaneously improve access and retention in care and also address health and social needs, communities and peers may need to be empowered to take an active role [47]. The National HIV Strategic Plan recommends such strategies to be implemented in Myanmar [22].

Our results show that PLHIV with higher viraemic-time should benefit from differentiated care, including more frequent clinical visits, better adherence support, HIV drug-resistance monitoring, and eventually an early switch to third-line treatment when resistance to second-line drugs emerges. Additionally, the periodicity of VL monitoring may need to increase. In 2015, Marks et al. demonstrated that when VL monitoring was done less frequently, patients were at risk for longer periods with viraemia. They argued that more frequent virological monitoring might reduce viraemic-time, transmission risk, and improve individual health of PLHIV [30]. In the future, novel tools that allow point-of-care VL testing will revolutionise HIV control [48]. Meanwhile, subgroups at risk for higher viraemic-time should be prioritized for more frequent VL monitoring. In our study cohort with access to frequent routine VL monitoring, 40% of PLHIV experienced viraemia while being on second-line ART. Only a minority of PLHIV were switched to third-line ART because only confirmed HIV drug resistance was an indication for treatment change. Indeed, HIV drug-resistance testing is recommended to distinguish between viraemia caused by treatment failure due to suboptimal adherence and treatment failure due to HIV drug resistance. For the first type of patients, third-line ART is not needed and may even cause harm, as in the long run a precious treatment option might be lost. Only for the latter group, a switch to third-line ART is justified. As access to drug-resistance testing as part of routine clinical care and/or access to third-line medications remain very limited, many patients continue second-line treatment despite viraemia [49]. Hermans et al. have recently recommended using ART-drug serum-level measurements as a strategy to differentiate causes of treatment failure and accelerate clinical decision making [50]. An early switch to effective treatment after first-line treatment failure was modelled to be a lifesaving opportunity, especially for those with advanced HIV disease [51]. Similar studies should assess if the same approach would benefit those with second-line treatment failure, keeping in mind increased HIV transmission risk.

PLHIV on a lopinavir/ritonavir-based regimen experienced higher viraemic-time in our cohort, consistent with previously reported findings [52]. Long-term previous exposure to first-line ART was not associated with a higher viraemic-time once patients were on second-line treatment. However, routine virological monitoring for patients on first-line ART in our cohort was introduced only after 2016. In patients with first-line virological failure were and not switched in time resistance accumulates to the NRTIs [53], the backbone of second-line ART regimens. This leads to a less effective second-line regimen and more-frequent second-line failures [54, 55]. To overcome such challenges in the future, there is a need to scale up access to relatively new antiretrovirals, such as dolutegravir or darunavir/cobicistat, known to be safe and highly effective, and at the same time to have a higher genetic barrier and longevity in comparison with currently used treatments [56–59].

Our study identified other hazards for mortality among PLHIV on second-line ART. Our results show an increase in mortality hazard for every year increase in age. The older, the higher risk of NCDs or some type of opportunistic infections. However, careful mortality ascertainment was not done and known causes of death among adults were not included in the regression model. PLHIV who received lopinavir/ritonavir had a higher risk of mortality when compared to those on atazanavir/ritonavir. The association between the type of protease inhibitor and mortality is probably not related to the drug itself, but the underlying indication for its use. Unfortunately, data on such indications (e.g. an episode of tuberculosis during second-line treatment) were not included in the study.

Our study represents the reality of a large long-term HIV programme in the Southeast Asian context with an exceptionally high coverage of routine VL testing. However, there are several limitations. Sampling bias caused by irregular VL testing has been discussed as a limitation of cumulative indicators of viraemia [60]. In our study, only a minority of VL tests were more than six months delayed. Moreover, we limited time allocation to a VL result to a maximum of 365 days, with a maximum of 183 days before and 183 days after the date of VL testing. Assessment of sensitive information, such as sexual preferences (gay men or men having sex with men), involvement in sex work, and injecting drug status was done at baseline only. Possible changes over time were not recorded. Furthermore, reporting bias might have occurred as PLHIV in this setting tend to underreport risk behaviour due to stigma and criminalisation of these activities. This may have resulted in an underestimation of the frequency of such risk behaviours and may have affected their association with outcomes. Because treatment adherence was not recorded in the database it could not be assessed as a predictor. This may have caused residual confounding. The study includes patients from Myanmar and is unlikely to be representative of geographically different groups. Conversely, findings are likely generalizable to other second-line ART cohorts in Myanmar, given that the MSF HIV programme included a quarter of the estimated total population of PLHIV in the country.

## Conclusions

Among PLHIV on second-line ART in Myanmar, having less than 50% viraemic-time was not associated with mortality but could be considered as a risk factor for ongoing HIV transmission, especially among key populations. Viraemic-time >50% was associated with mortality. Cross-sectional analysis of VL data does not allow clinicians and programme managers to assess the clinical and public health impact of higher viraemic-time. Cumulative assessments of HIV viraemia might better reflect the health status and the level of HIV control of the entire cohort than cross-sectional indicators. To reduce the risk of HIV transmission and mortality among those who are failing second-line HIV treatment, differentiated services including innovative approaches for the management of viraemia and models of care tailored to the specific needs of at-risk groups should be scaled up.
