# Correction: Viraemic-time predicts mortality among people living with HIV on second-line antiretroviral treatment in Myanmar: A retrospective cohort study

**DOI:** 10.1371/journal.pone.0299460

**Published:** 2024-02-22

**Authors:** Anita Mesic, Tom Decroo, Htay Thet Mar, Bart K. M. Jacobs, Moe Pyae Thandar, Thin Thin Thwe, Aung Aung Kyaw, Mitchell Sangma, David Beversluis, Elkin Bermudez-Aza, Alexander Spina, Darli Po Po Aung, Erwan Piriou, Koert Ritmeijer, Josefien Van Olmen, Htun Nyunt Oo, Lutgarde Lynen

There are errors in the Funding and Competing Interests statements. The correct statements are:

**Funding:** MSF provided financial support for this study. The HIV programme was financed under the operational cost of MSF Myanmar mission, and MSF provided support for the publication costs. MSF also provided support for this study in the form of salaries for AM, HTM, MPT, TTT, AAK, MS, DB, EBA, AS, EP, and KR. The specific roles of these authors are articulated in the ‘author contributions’ section. MSF had a role in study design, data collection and analysis, decision to publish and preparation of the manuscript. This is indicated by the affiliation of the co-authors and their role in the study and publication.[1]

**Competing Interests**: The authors have read the journal’s policy and have the following competing interests to declare: MSF provided support for this study in the form of salaries for AM, HTM, MPT, TTT, AAK, MS, DB, EBA, AS, EP, and KR. This does not alter our adherence to *PLOS ONE* policies on sharing data and materials. There are no patents, products in development or marketed products associated with this research to declare.
